# Agent-based modeling of the prostate tumor microenvironment uncovers spatial tumor growth constraints and immunomodulatory properties

**DOI:** 10.1038/s41540-024-00344-6

**Published:** 2024-02-21

**Authors:** Maisa N. G. van Genderen, Jeroen Kneppers, Anniek Zaalberg, Elise M. Bekers, Andries M. Bergman, Wilbert Zwart, Federica Eduati

**Affiliations:** 1 Department of Biomedical Engineering, Eindhoven University of Technology, PO Box 513, 5600MB Eindhoven, The Netherlands; 2 Division of Oncogenomics, Oncode Institute, Netherlands Cancer Institute, Plesmanlaan 121, 1066 CX Amsterdam, The Netherlands; 3 Division of Pathology, Netherlands Cancer Institute, Plesmanlaan 121, 1066 CX Amsterdam, The Netherlands; 4 Division of Medical Oncology, Netherlands Cancer Institute, Plesmanlaan 121, 1066 CX Amsterdam, The Netherlands; 5 Institute for Complex Molecular Systems, Eindhoven University of Technology, PO Box 513, 5600MB Eindhoven, The Netherlands

**Keywords:** Cancer, Systems biology, Oncology

## Abstract

Inhibiting androgen receptor (AR) signaling through androgen deprivation therapy (ADT) reduces prostate cancer (PCa) growth in virtually all patients, but response may be temporary, in which case resistance develops, ultimately leading to lethal castration-resistant prostate cancer (CRPC). The tumor microenvironment (TME) plays an important role in the development and progression of PCa. In addition to tumor cells, TME-resident macrophages and fibroblasts express AR and are therefore also affected by ADT. However, the interplay of different TME cell types in the development of CRPC remains largely unexplored. To understand the complex stochastic nature of cell-cell interactions, we created a PCa-specific agent-based model (PCABM) based on in vitro cell proliferation data. PCa cells, fibroblasts, “pro-inflammatory” M1-like and “pro-tumor” M2-like polarized macrophages are modeled as agents from a simple set of validated base assumptions. PCABM allows us to simulate the effect of ADT on the interplay between various prostate TME cell types. The resulting in vitro growth patterns mimic human PCa. Our PCABM can effectively model hormonal perturbations by ADT, in which PCABM suggests that CRPC arises in clusters of resistant cells, as is observed in multifocal PCa. In addition, fibroblasts compete for cellular space in the TME while simultaneously creating niches for tumor cells to proliferate in. Finally, PCABM predicts that ADT has immunomodulatory effects on macrophages that may enhance tumor survival. Taken together, these results suggest that AR plays a critical role in the cellular interplay and stochastic interactions in the TME that influence tumor cell behavior and CRPC development.

## Introduction

Prostate cancer (PCa) is the second most common cancer in men worldwide, with 1.4 million new cases and over 370,000 deaths annually^1^. Androgen receptor (AR) signaling plays a pivotal role in PCa initiation and progression, motivating the development of several therapies targeting this hormone-driven transcription factor over the years^2–4^. However, despite an initial treatment response in most patients, resistance to androgen deprivation therapy (ADT) inevitably develops, resulting in lethal metastatic castration-resistant prostate cancer (CRPC)^5^. Therefore, the development of new therapies that effectively treat or even prevent CRPC is critical^6^.

Recently, multiple studies have shown that the tumor microenvironment (TME) plays a key role in the development and progression of PCa^7–11^. The prostate TME consists of a variety of non-malignant cells, including fibroblasts and macrophages^12–15^. Cells in the TME influence PCa cell growth through chemical and physical interactions between tumor- and stromal cells, through angiogenesis, immune suppression, extracellular matrix (ECM) remodeling and tumor invasion^10,16–18^. Although fibroblasts are mostly quiescent in healthy tissues, in the TME fibroblasts are in a state reminiscent of wound healing and are referred to as cancer-associated fibroblasts (CAFs)^12,19,20^. Another dominant component of the prostate TME is macrophages, which are highly plastic cells that can polarize into a spectrum of phenotypes. Conventionally, two extreme polarizations of tumor-associated macrophages are recognized: classically activated pro-inflammatory (M1) macrophages and alternatively activated anti-inflammatory (M2) macrophages^21,22^. In general, M1-macrophages are anti-tumorigenic leading to tumor cell death, whereas M2-like macrophages are pro-tumorigenic, promoting tumor growth. These phenotypically distinct macrophages have been hypothesized to have contrasting effects on tumor progression^23^. Importantly, specific macrophage subtypes have a prognostic value for PCa patients, suggesting that the relative contributions of these subtypes are related to patient outcome^24^.

Interestingly, AR expression is not restricted to PCa cells, but is also expressed and functional in cells of the prostate TME, including fibroblasts and macrophages^25^. Consequently, interactions between cells of the prostate TME could potentially be affected by androgens and thus by AR-targeted therapies, including ADT. However, studies on ADT altering TME cell interactions in the context of primary PCa and CRPC development are limited and present conflicting results. Low levels of AR in stromal tissues are associated with an earlier onset of PCa recurrence^8,26^. Indeed, AR signaling in the stroma has been reported to play a protective role in PCa development, as low AR expression in the TME is associated with a high-grade tumor and poor clinical outcome^8^. Previously, we have shown that AR inhibition in CAFs triggers PCa cell migration via paracrine regulation of CCL2 and CXCL8, which may contribute to PCa invasiveness and metastasis^26^. Alternatively, infiltration of tumor-associated macrophages (TAMs) influences disease progression toward CRPC development after ADT^27–29^. AR signaling in macrophages activates TREM-1 signaling, which subsequently leads to the secretion of pro-inflammatory cytokines that support PCa cell line migration and invasion^30^. In addition, AR has been described as an enhancer of macrophage and monocyte differentiation^31,32^. However, it is not fully understood how the combined interactions between TME cells contribute to CRPC development and what the role of ADT is in these interactions.

Over the years, computational models have emerged as powerful tools in the field of prostate cancer research, offering valuable insights into various facets of the disease. From elucidating the intricate mechanics governing prostate cancer growth^33^ to employing ordinary or stochastic differential equations to model the dynamic interplay between different cell populations in response to therapy^34–39^, computational modeling has significantly contributed to our understanding of this complex malignancy. However, existing models often neglect the spatial component, failing to capture individual cell interactions within the TME. Addressing this gap, agent-based models (ABMs) provide a complementary approach by representing cells as autonomous agents, allowing for a detailed examination of spatial dynamics^40^. With ABMs it is possible to model individual agents that perform stochastic actions, thereby creating complexity from a simple set of base cell actions. Previously, ABMs have been successfully applied to study tumor stem cell growth^41,42^, tumor cell migration^43^, avascular tumor growth^44^, radiotherapy optimization^45^ and response to immunotherapy in colorectal cancer^46,47^. Recently we developed an ABM to study prostate cancer onset, however this does not account for the effect of therapy on the prostate tumor microenvironment^48^.

In this study we generated a PCa-specific ABM (PCABM) which includes the interactions between tumor cells, fibroblasts, and macrophages in relation to hormonal therapy. The PCABM is informed by in vitro prostate TME co-culture growth data, using particle swarm optimization (PSO). PCABM simulations show that CRPC is multifocal and arises from clusters of resistant cells within the prostate TME. In addition, fibroblasts play an indispensable role in regulating spatial proliferative constraints while simultaneously providing a protective niche for tumor cells from the tumoricidal effect of pro-inflammatory macrophages. Finally, PCABM suggests that ADT may have immunomodulatory effects on the prostate TME, impacting macrophage-mediated tumor cell killing in androgen deprived conditions, leading to possible PCa cell growth after ADT.

Cumulatively, our in silico model faithfully phenocopies both the response of tumor cells to hormonal stimuli, as well as the impact of therapy thereon in relation to its microenvironment.

## Results

### PCABM conceptual model

**Fig. 1:**
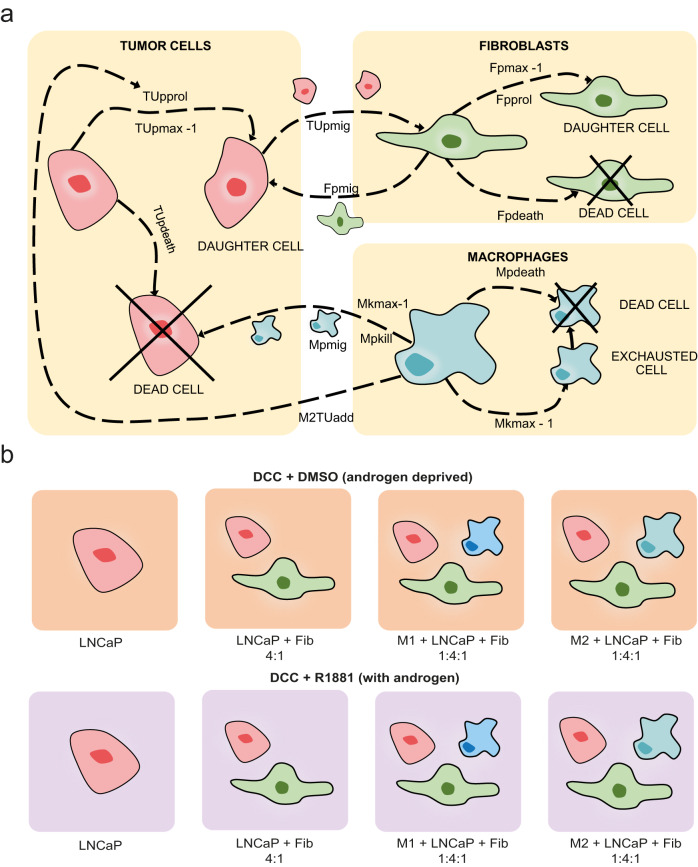
PCABM parameter and cell type action overview. **a** Overview of all modeled cell interactions, in which each cell type can migrate, idle and die. Tumor cells and fibroblasts proliferate, while macrophages can either kill or support tumor cells depending on their subtype. **b** PCABM is optimized for two in vitro co-culture conditions: cells grown in dextran coated charcoal (DCC) supplemented medium without androgen (DMSO, upper panels) and with androgen (R1881, lower panels). The different cell types are LNCaP, LNCaP + fibroblasts and LNCaP + fibroblasts + either M1 or M2-polarized macrophages.

We developed an ABM consisting of tumor cells, fibroblasts, M1 and M2 macrophages, which are seen as agents and scattered randomly on grid upon initialization to mimic in vitro settings. These cellular agents perform actions (proliferate, die) and interact with each other as schematically represented in Fig. 1a (see Methods for a more extensive description of the model).

Using particle swarm optimization (PSO), we optimized the PCABM on co-cultures experimental data (six technical replicates spanning three biological replicates) measured in androgen proficient R1181 conditions versus hormone deprived vehicle control conditions to mimic the TME in normal and ADT conditions respectively (Fig. 1b, experimental co-culture growth curves are shown in Supplementary Fig. 1). Model parameters are reported in Table 1 (see Methods for more details on the parameter estimation procedure).

**Table 1:** Overview of parameters

Parameter	Description	DCC DMSO	DCC R1881	Source	Optimization
TUpprol	Probability of tumor cell proliferation	0.0389	0.1144	Own data	PSO
TUpmig	Probability of tumor cell migration	0.1	0.1167	45 & own data	
TUpdeath	Probability of tumor cell death	0.00248	0.00248	Own data	PSO
TUrwalk	Random influence on tumor cell movement	0.5	0.5	Own data	Qualitative
TUpmax	Proliferation capacity of tumor cells	4	4	Own data	PSO
TUpres	Probability of tumor cell becoming resistant upon proliferation	0.002	0	Own data	PSO
TUpprolres	Probability of resistant tumor cell proliferation	0.0596	0	Own data	PSO
TUpmigres	Probability of resistant tumor cell migration	0.1167	0	Own data	PSO
TUpmaxres	Proliferation capacity of resistant tumor cell	50	0	Own data	PSO
M1kmax	Killing capacity of M1 macrophage	11	11	Own data	PSO
M1pkill	Probability of M1 macrophage killing adjacent tumor cell	0.005	0.1116	Own data	PSO
M1pmig	Probability of M1 macrophage migration	0.2667	0.2667	46	
M1rwalk	Random influence on M1 macrophage movement	0.8	0.8	46	
M1speed	Speed of M1 macrophage movement	40	40	46	
M1engagementDuration	Number of steps M1 macrophage is engaged in killing tumor cell	60	60	46	
M2kmax	Killing capacity of M2 macrophage	11	11	Own data	PSO
M2pkill	Probability of M2 macrophage killing adjacent tumor cell	0.0348	0.0223	Own data	PSO
M2pmig	Probability of M2 macrophage migration	0.2667	0.2667	46	
M2rwalk	Random influence on M2 macrophage movement	0.8	0.8	46	
M2speed	Speed of M2 macrophage movement	40	40	46	
M2engagementDuration	Number of steps M2 macrophage is engaged in killing tumor cell	60	60	46	
M2TUadd	Addition to proliferation probability of tumor cells if M2 macrophages are present in the system.	0	0.0995	Own data	PSO
Fpprol	Probability of fibroblast proliferation	0.0838	0.0838	Own data	PSO
Fpmig	Probability of fibroblast migration	0.4	0.4	Own data	Qualitative
Fpdeath	Probability of fibroblast death	0.0018	0.0018	Own data	PSO
Frwalk	Random influence on fibroblast movement	0.5	0.5	Own data	Qualitative

### PCABM forms similar growth patterns as in vitro co-cultures and histological samples

**Fig. 2:**
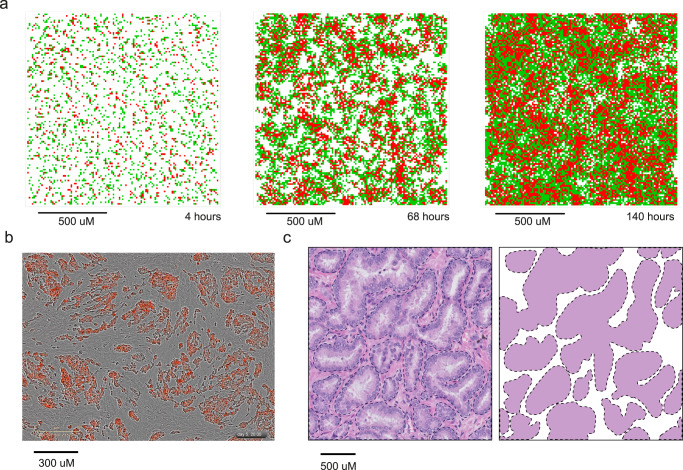
Prostate TME spatial patterns in silico, in vitro, and in vivo in hormone proficient conditions. **a** Modeled tumor cells (red) and fibroblasts (green) are randomly distributed across PCABM lattice, but spatiotemporally organize after 4, 68 and 140 h of pseudo-time (1:1 ratio). **b** In vitro co-culture of tumor cells (red) and fibroblasts (brightfield, 1:1 ratio) after 140 h. **c** FFPE H&E staining at 200x magnification of a primary prostate tumor, showing distinct epithelial tumor foci surrounded by stroma (left), with masked image of these foci (right).

Upon initialization of PCABM, cells are randomly distributed across a grid and self-organized to form complex spatial patterns over time (Fig. 2a). In our in silico PCABM, we observe similar spatial growth patterns to those observed in vitro (Fig. 2b) and to those observed in human tumor samples, as identified in hematoxylin and eosin (H&E) stained formalin-fixed paraffin embedded prostate tumor tissue (Fig. 2c). Specifically, in all settings we observe foci of tumor cells surrounded by fibroblasts or stroma on similar spatial scales. These observations illustrate PCABM’s ability to reliably model spatial PCa growth pattern complexity in silico from a simple set of assumptions and optimizations.

### Hormonal response of PCa cells is accurately captured by PCABM

**Fig. 3:**
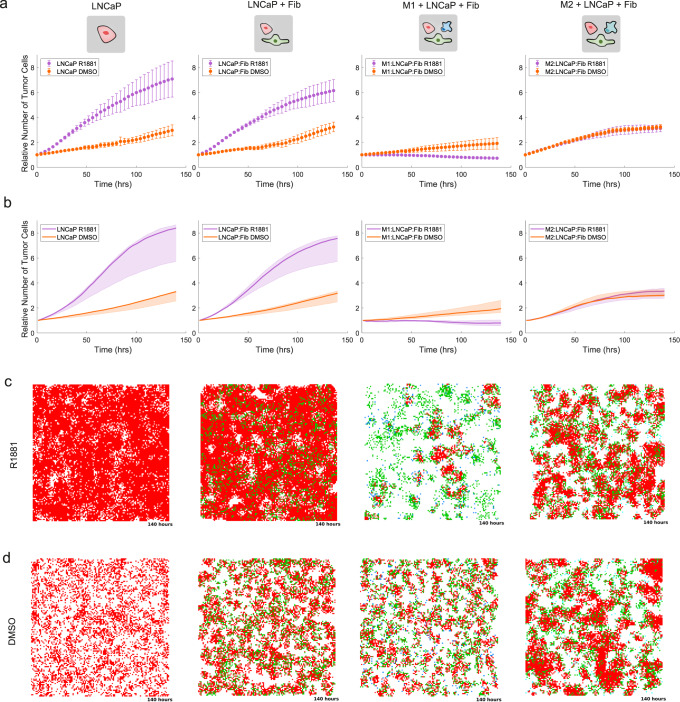
In vitro tumor cell proliferation and hormone response is accurately captured by PCABM’s optimized in silico parameters. **a** Incucyte data for different co-cultures in hormone deficient (DMSO, orange) and hormone proficient (R1881, purple) conditions for sequentially LNCaP monoculture; LNCaP and fibroblast co-culture (4:1 ratio); LNCaP, fibroblast and M1-polarized macrophage co-culture (4:1:1 ratio) ; LNCaP, fibroblast and M2-polarized macrophage co-culture (4:1:1 ratio). **b** PCABM model behavior after parameter optimization in hormone deficient (DMSO, orange) and hormone proficient (R1881, purple) in silico conditions for sequentially LNCaP monoculture; LNCaP and fibroblast co-culture (4:1 ratio); LNCaP, fibroblast and M1-polarized macrophage co-culture (4:1:1 ratio); LNCaP, fibroblast and M2-polarized macrophage co-culture (4:1:1 ratio). **c** Spatial maps of in silico end points with different parameter sets in hormone proficient (R1881) conditions for sequentially LNCaP monoculture; LNCaP and fibroblast co-culture (red, green; 4:1 ratio); LNCaP, fibroblast and M1-polarized macrophage co-culture (red, green, dark blue; 4:1: ratio); LNCaP, fibroblast and M2-polarized macrophage co-culture (red, green, light blue; 4:1:1 ratio). **d** Spatial maps of in silico end point with different parameter sets in hormone deficient (DMSO) conditions for sequentially LNCaP monoculture; LNCaP and fibroblast co-culture (red, green; 4:1 ratio); LNCaP, fibroblast and M1-polarized macrophage co-culture (red, green, dark blue; 4:1: ratio); LNCaP, fibroblast and M2-polarized macrophage co-culture (red, green, light blue; 4:1:1 ratio). Data represent the average of three biological replicates, with six technical replicates each. Error bars indicate standard deviation. Lines represent PCABM model output with the median of optimized parameters over three biological replicates. Shading represents model output for optimized parameters within interquartile range given by 50 optimizations for each biological replicate.

PCABM simulations recapitulate LNCaP cell growth curves observed in in vitro experiments well in both hormone proficient and deficient conditions (Fig. 3). Model estimation of tumor cell proliferation (TU_pprol_) shows a threefold increase in tumor cell proliferation as response to R1881 treatment (TU_pprol_ = 0.1144 for R1881 versus 0.0389 vehicle control; Supplementary Fig. 2 for parameter optimizations). When adding fibroblasts in silico to the culture under R1881 conditions, a slight reduction in the growth rate is observed without changing proliferation parameters, matching the corresponding experimental data (Fig. 3). This change underlines the predictive power for ABM stochastic modeling without additional adjustments.

Co-culturing M1-polarized macrophages together with LNCaP and fibroblast, we observed an in vitro strong decrease in tumor growth rate compared to LNCaP mono-cultures and LNCaP + fibroblast co-cultures, while such an effect was less apparent in the hormone deprived condition (Fig. 3a). By simulating the same experimental condition (i.e. model with LNCaP, fibroblasts and M1 macrophages) and optimizing PCABM’s M1 macrophage killing probability (M1_pkill_) based on these data, we found a 22-fold decrease in killing capacity in hormone deficient (DCC + DMSO) versus hormone proficient (DCC + R1881) conditions (M1_pkill_ = 0.005 and 0.1116 respectively; Fig. 3b and Supplementary Figs. 3, 4). In contrast, replacing M1-like for M2-like polarized macrophages did not result in a differential effect in growth curves between hormone conditions both in vitro and in silico (Fig. 3a, b). Such cell culture growth dynamics could be reliably reproduced in silico using PCABM, with different observed tumor cell proliferation and kill capacities in the hormonal conditions for M2-polarized macrophages (TU_pprol_ R1881 = 0.0389 and TU_pprol_ DMSO = 0.1348; M2_pkill_ R1881 = 0.0223, M2_pkill_ DMSO = 0.0348; Fig. 3b, Supplementary Figs. 3, 4). In silico spatial patterns in hormone proficient versus deficient conditions (Fig. 3C and D respectively) recapitulate these changes in proliferation and macrophage killing capacity. Taken together, these data suggest that PCABM accurately describes PCa cell proliferation potential and the impact of R1881 treatment thereon, when co-cultured with different TME cell types.

### PCABM predicts immunomodulatory effects of ADT on macrophages

**Fig. 4:**
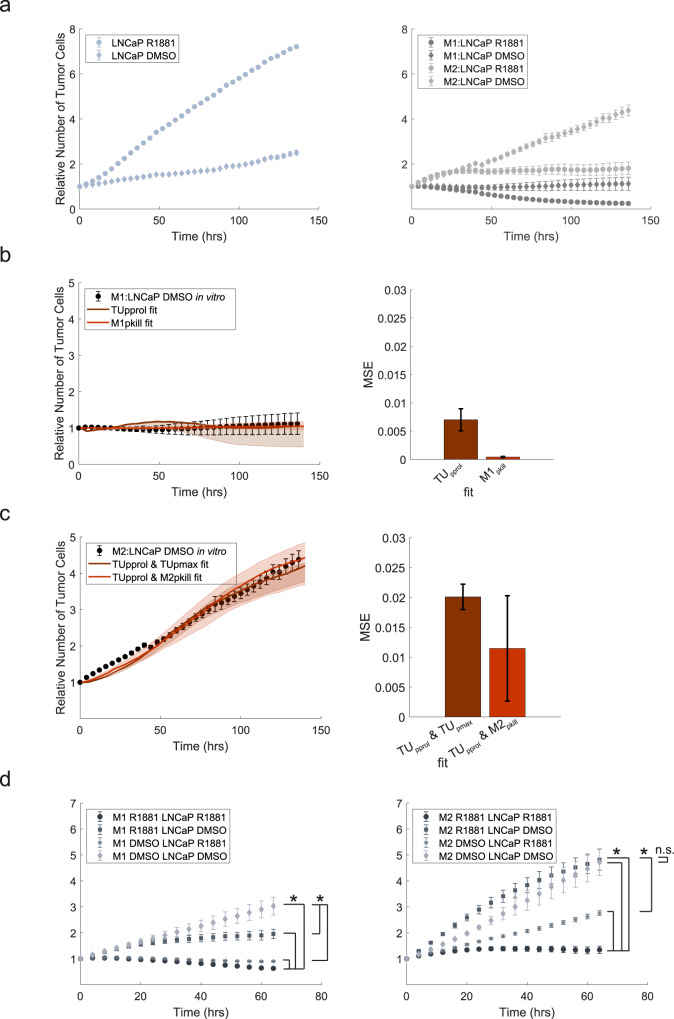
PCABM predicts immunomodulatory ADT-mediated macrophage tumoricidal effects. **a** LNCaP growth curve alone (left) or in co-culture with M1- or M2-macrophages (right) in absence or presence of R1881. **b** PCABM optimization for TU_pprol_ and M1_pkill_ in DCC + DMSO (left) and mean squared error (MSE) between experimental data and PCABM for M1:LNCaP TU_pprol_ and M1_pkill_ (right). **c** PCABM optimization for TU_pprol_ and or M2_pkill_ in DCC DMSO (left) and mean squared error (MSE) between experimental data and PCABM for M2:LNCaP TU_pprol_ + TU_pmax_ and TU_pprol_ + M2_pkill_ (right). **d** Growth curve of LNCaP co-cultured with M1-macrophages individually stimulated with DMSO or R1881 (left) and growth curve of LNCaP co-cultured with M2-macrophages individually stimulated with DMSO or R1881 (right). Bars and error represent mean and standard deviation over MSE of 50 optimizations for replicate 1. Dots represent average and error bars represent standard deviation of six technical replicates. Lines represent PCABM output with the median of optimized parameters. Shading represents model output for optimized parameters within interquartile range given by 50 optimizations.

Through PCABM parameter optimization we further estimated whether the hormone-driven decrease of LNCaP cell growth in co-culture with M1 or M2 polarized macrophages was tumor cell intrinsic or related to macrophage tumoricidal activity. For this purpose, we cultured LNCaPs with macrophages but without the presence of fibroblasts and saw differences compared to previous growth rates, with a clear tumoricidal effect for M1 macrophages supplemented with R1881 (Fig. 4a). Paradoxically, optimizing LNCaP TU_pprol_ in vehicle conditions while using macrophage M1_pkill_ and M1_kmax_ that we previously optimized in hormone-proficient conditions, resulted in higher predicted proliferation values (TU_pprol_ DMSO = 0.1550; TU_pprol_ R1881 = 0.1144, Fig. 4b, Supplementary Fig. 4). Since higher LNCaP TU_pprol_ is expected upon R1881 treatment, we optimized M1_pkill_ while keeping LNCaP proliferation constant on vehicle conditions (TU_pprol_ DMSO = 0.0389), which resulted in an improved PCABM fit to in vitro data with smaller mean square error (MSE) between data and model fit for all three in vitro replicates (Fig. 4b, Supplementary Fig. 5). Importantly, R1881 conditions increased M1_pkill_ capacity 21–46 fold (M1_pkill_ DMSO = 0.005 in vehicle control; M1_pkill_ R1881 = 0.2034). These PCABM optimizations suggest that changes in tumor cell viability upon hormone deprivation are not solely dictated by decreased tumor cell proliferation but are also impacted by M1 macrophage tumoricidal effects.

To observe whether such an approach would also improve MSEs in the M2-polarized PCABM, and whether M2-macrophage polarization has differential effects on the TME compared to M1-polarized macrophages, we again optimized M2_pkill_ while keeping LNCaP proliferation constant to vehicle conditions (TU_pprol_ DMSO = 0.1341), with TU_pmax_ DMSO = 5, which only slightly improved PCABM fit and MSEs (Fig. 4c, Supplementary Fig. 5). As expected, PCABM indicates that M2-macrophages exhibit less tumoricidal activity compared to M1-macrophages and become tumor promoting in vehicle conditions, enhancing predicted tumor growth (TU_pprol_ 2-3 fold increase) while decreasing tumor killing capacity (M2_pkill_ 2–4 decrease) relative to R1881 conditions (TU_pprol_ DMSO = 0.0384 and TU_pprol_ R1881 = 0.1128 and M2_pkill_ DMSO = 0.0219 and M2_pkill_ R1881 = 0.0441; Fig. 4c, Supplementary Table 1). In co-cultures, we validated these findings with individually stimulated co-culture cell constituents. For M1 co-cultures we observed that growth is significantly increased in hormone deprived conditions, while for M2 co-cultures this effect is not present (Fig. 4d). These results suggest that ADT exerts an immunomodulatory effect on tumor cell killing.

### Spatial effects in the TME and differential macrophage tumoricidal capacities enhance TME cellular dynamics

**Fig. 5:**
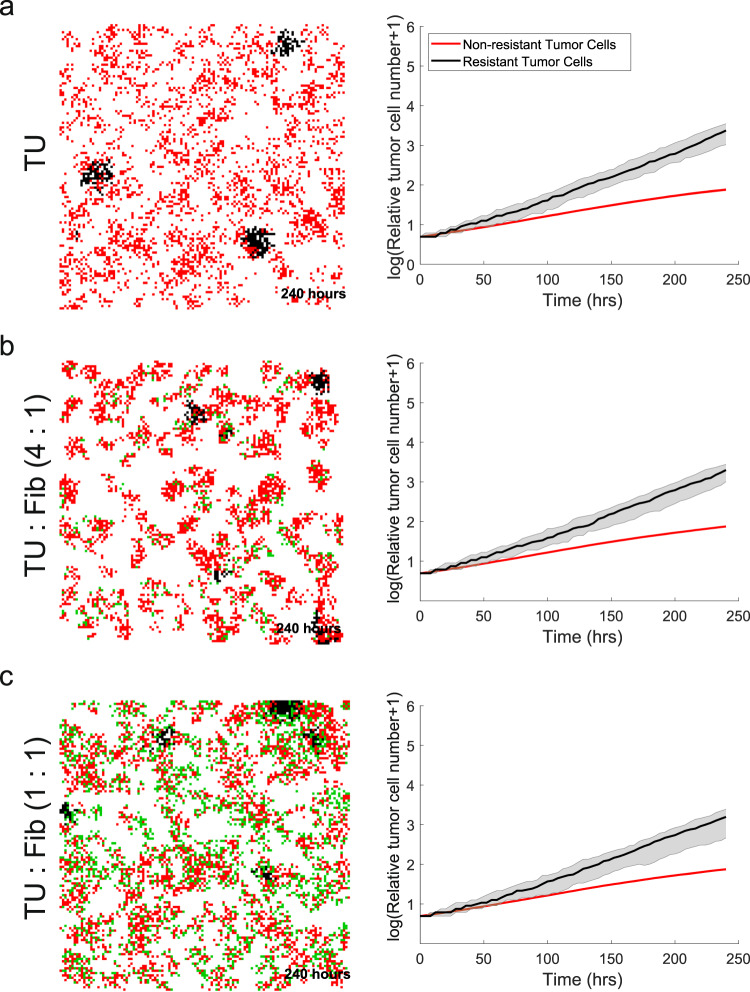
CRPC simulations in PCABM with fibroblasts. **a** Growth of LNCaP and LNCaP-abl cells. **b** Relative growth of tumor cells seeded with fibroblasts at a 4:1 ratio. **c** Relative growth of tumor cells seeded with fibroblasts at a 1:1 ratio. For all panels, PCABM (left) is compared to Incucyte (right) of co-cultures of LNCaP cells with LNCaP-abl cells and fibroblasts.

We next sought to investigate how the TME contributes to the emergence of CRPC. Experimental data from castration resistant LNCaP-abl (androgen ablated) cells grown in hormone deprived conditions was used to fit proliferation parameters for resistant cells (Supplementary Fig. 2C). In contrast to LNCaP cells, in vitro LNCaP-abl growth increases exponentially in hormone deprived conditions. Therefore, to mimic LNCaP-abl growth observed in vitro, we optimized a higher tumor cell proliferation (TU_pprolres_ = 0.06) for resistant cells, which is almost twice that of LNCaP TU_pprol_ in hormone deprived conditions. Interestingly, LNCaP-abl cells readily form clusters of resistant cells in silico (Fig. 5a and Supplementary Fig. 6), which is also observed when growing LNCaP-abl cells in vitro (Supplementary Fig. 7). These cluster formations were robust to slight changes in resistant cell parameters (Supplementary Fig. 8).

While the in silico addition of fibroblasts does not affect proliferation speed, there are increased fibroblast directional migration effects towards tumor cells. These effects result in increased hormone-sensitive tumor-cell cluster formation, which in turn is balanced by cellular competition for space as fibroblasts take up growth space (Fig. 5b, c). These data suggest that not only the population growth of TME constituents, but that also the available TME space is an important characteristic to describe the entirety of TME cellular growth dynamics.

### Macrophage phenotype and influx play a critical role on resistant tumor cell growth

**Fig. 6:**
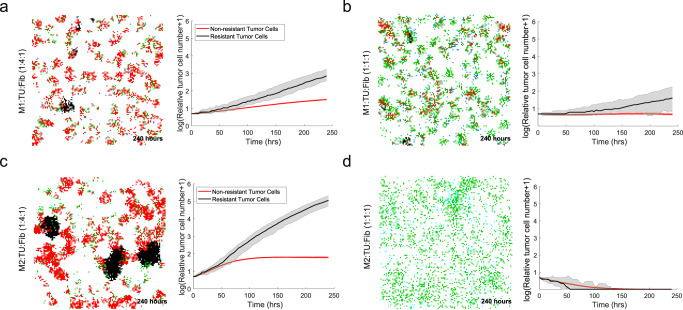
CRPC simulations in PCABM with fibroblasts and either M1 and M2 polarized macrophages. **a** M1 macrophages seeded with tumor cells and fibroblasts at a 1:4:1 ratio. **b** M1 macrophages seeded with tumor cells and fibroblasts at a 1:1:1 ratio. **c** M2 macrophages seeded with tumor cells and fibroblasts at a1:4:1 ratio. **d** M2 macrophages seeded with tumor cells and fibroblasts at a 1:1:1 ratio. For all panels, PCABM (left) is compared to Incucyte (right) of co-cultures of LNCaP cells and LNCaP-abl cells, fibroblasts and M1- or M2-polarized macrophages.

Next, we further enriched our in silico model, by including tumoricidal M1 polarized macrophages in CRPC-PCABM, which has a repressing effect on both CRPC and hormone responsive PCa proliferation speed. Since the number of tumor-resident macrophages vary greatly between PCa samples^24,49^ which can be partially explained by differences in tumor volume and macrophage influx, we wondered how PCABM would respond to varying levels of macrophages. When quadrupling the amount of macrophages to tumor cells, tumor cell population extinction is quickly achieved in silico (Fig. 6a, b). Interestingly, the addition of a large fibroblast presence seems to reduce macrophage tumoricidal effects (Fig. 6b). Conversely, M2-polarized macrophages significantly increase tumor cell proliferation, as shown experimentally in a co-culture with colorectal cancer cells and M2-macrophages and described by others also in the context of PCa^50–55^.The increase in tumor cell proliferation is more pronounced in CRPC as compared to hormone-sensitive PCa cells (Fig. 6d, e). Additionally, when changing the ratios between tumor cells and M2-polarized macrophages we observe a growth reduction of both resistant and hormone-sensitive tumor cells (Fig. 6d, e, also see Supplementary Fig. 6 for additional conditions). Taken together, these observations demonstrate how a higher influx of macrophages lead to tumor remission even in the context of resistant tumor cells, while fibroblasts provide a protective niche for resistant tumor cells to proliferate in.

## Discussion

Because AR plays a key role in PCa progression, patients with metastatic disease recurrence are typically treated with AR-targeted therapeutics^56,57^. Since cells in the TME also express AR, they are consequently also affected by ADT, which could affect cell-cell interactions. In this work, we replicated ADT-conditions in silico in a PCa-specific ABM, which is able to model the spatiotemporal complexity of prostate TME cell interactions in both hormone pro- and deficient conditions. By implementing a simple set of stochastic assumptions, an intrinsically organized, self-assembling TME cellular structure emerges in PCABM that resembles the histology in PCa patient samples. Since PCa is multifocal in 60–90% of cases^58^, these simulated tumor foci further underscore the ability of the PCABM to form potentially clinically relevant spatial patterns and suggest that the TME could play a critical role in the formation of multifocal disease, although testing in more in vivo-like model systems would be required to validate this hypothesis.

Our modeling assumptions were calibrated and refined using data from extensive in vitro co-cultures, that incorporate cell proliferation and migration data. Because PCABM is currently modeled only for LNCaP cells, it is limited in its ability to accurately replicate PCa growth and development of CRPC. However, the model is adaptable to other AR-positive PCa cell lines, provided that in vitro data exists for calibration demonstrating its strength in that the parameters are easily adaptable to other hypotheses. Multiple PCa cell lines have been developed with a wide variety of proliferation kinetics and response to hormones, which may lead to different PCABM results. Recently, we reported a genome-wide CRISPR screen in PCa cells co-cultured with pro-inflammatory macrophages where we identified AR as a critical regulator of macrophage-mediated killing^59^. These studies revealed AR as a genuine tumor-intrinsic immunomodulator, with hormone deprivation preventing tumor cell killing by M1 macrophages. Fully in line with this, our PCABM predicts that ADT affects the cellular behavior of both tumor cells and M1 macrophages, further solidifying our observation that AR plays an immunomodulatory role in the prostate TME. Independent in vitro experiments validated this, suggesting that ADT affects the differentiation of this cell type, which may potentially stimulate tumor growth. Interestingly, the addition of fibroblasts to PCABM stimulates directional migration of both tumor cells and fibroblasts, resulting in a limited amount of space around the tumor cells. In androgen proficient conditions such a proliferation space will be severely limited due to high proliferation rates, whereas in androgen deficient conditions, such an effect will theoretically be less pronounced due to decreased proliferation rates of AR-responsive cells. These results suggest that fibroblasts block the access of M1 macrophages to tumor cells by their preferential clustering around tumor cells. Since macrophages are able to kill tumor cells through cell-to-cell contact^46^, fibroblasts may prevent macrophages from completing their tumoricidal activity.

In addition, we modeled CRPC formation in PCABM and showed that resistant cells form separate clusters due to the directional migration effects of fibroblasts. These findings support the multifocality of PCa and further highlight the tumor-protective role of fibroblasts by limiting the physical access of macrophages while creating a niche for tumor cells. Previously, the amount of stroma has been shown to be inversely correlated with recurrence-free survival, suggesting that stromal cells may protect tumor cells from being killed^60,61^. Supporting this, M1 macrophages decreased the growth of both androgen-sensitive and -insensitive PCa cells, whereas M2 macrophages allowed castration-resistant tumor cells to rapidly take over the TME. Recently, tumor-associated macrophages have been associated with PCa progression after ADT^13^ and the development of CRPC^9^, which is supported by our findings on the immunomodulatory effects of ADT and CRPC growth. These findings are also consistent with our recent report, in which we showed that AR signaling in macrophages plays a critical role in PCa migration and invasion through TREM-1 signaling and a concomitant upregulation of IL-10^30^. In contrast, when AR signaling is blocked in CAFs, PCa cells migrate under the influence of upregulated CCL2 and CXCL8 secretion^26^. These studies further underline the tumor-driving effects of the prostate TME induced by ADT, along with the differential intercellular interactions in this context.

Technically, PCABM has been calibrated to in vitro time scales and data. For more in vivo-like PCABM representations, longer timescales are needed, and the currently modeled timescales could be extended with long-term culture data, although long-term culture has practical limitations, for example overconfluency in co-cultures. We approximated the prostate TME by including tumor cells, fibroblasts and macrophages, which are the most abundant cell types in PCa^62^. However, our model was solely based on in vitro data and is thus missing some characteristics of in vivo TMEs, such as: 1) Physical differences leading to varying mechanobiology such as 2D versus 3D spatiality, cell plate surface adherence versus embedded in a cellular mesh, 2) Conditional differences in growth factor types and concentrations, nutrient availability and oxygenation. 3) Cellular differences in cell-cell adhesion^63^, intratumoral heterogeneity, angiogenesis, presence of endothelial cells and immune cells, especially CD4+/CD8+T-cells. Modifications should be made when modeling more in vivo-like behavior. We recently explored the use of ABM to simulate an in vivo-like behavior of onset and progression of PCa by including tumor cells being able to acquire mutations and grow in an ellipsoid formation representing the prostatic acinus^48^. However, our in vivo-like model does not allow yet to simulate therapy response and development of resistance which is the focus of the model presented in this paper. Future research efforts could be focused on the integration of the two models to study the effect of ADT, possibly in combination with other treatments, in more in vivo setting, although this is currently limited by the lack of availability of in vivo data to train and validate such models. As all model systems, including cell lines, are intrinsically an imperfect representation of clinical reality, we believe the responses as observed in our ABM should be interpretated qualitatively, when aimed to transfer these findings towards the clinic.

In conclusion, we present PCABM, an in silico tool that simulates and accurately describes the functional interplay between prostate TME cells in hormone proficient and ADT conditions and in the emergence of CRPC. Our findings suggest that targeting TME cell types may provide a novel avenue for the treatment of CRPC, as different TME cell types influence castration-resistant tumor cell growth. In future research, PCABM could be used to design targeting strategies involving the TME to achieve optimal anti-tumor efficacy, which may serve as a blueprint for implementation in other cancer types.

## Methods

### Cell culture and M1- and M2 macrophage differentiation

The prostate cancer cell lines LNCaP (ATCC CRL-1740) and LNCaP-abl (ATCC CVCL-4793) the monocytic cell line THP-1 (ATCC TIB-202) and immortalized foreskin fibroblast BJ cell line (CRL-2522) were cultured in RPMI-1640 (Gibco) supplemented with 10% fetal bovine serum (FBS, Sigma) and 1% penicillin-streptomycin (P/S, Gibco). For hormonal related experiments all cells were cultured in RPMI 1640 supplemented with 5% Dextran Coated Charcoal (DCC, Sigma) stripped-serum and 1% P/S 3 days before to the start of the experiment. AR was induced with 10 nM R1881 (Sigma) supplemented RPMI-DCC. Cell lines were kept at low passage and regularly tested mycoplasma negative. THP-1 cells were stimulated with either 100 ng/mL (for M1 macrophages) or 50 ng/mL (for M2 macrophages) of phorbol 12-myristate 13-acetate (PMA, Sigma) for 48 h, followed by 24 h in fresh 10%FBS-RPMI. M1-macrophages were differentiated by 24 h stimulation of 10 ng/mL lipopolysaccharide (LPS, Sigma) and 10 ng/mL interferon-*γ* (IFN-*γ*, Peprotech), while M2-macrophages were differentiated by 72 h stimulation with 20 ng/mL IL-4 (Peprotech) and 20 ng/mL IL-13 (Peprotech).

### Lentiviral vector and transduction

Lentivirus was generated in HEK293T cells cultured in 10% FBS, 1% P/S supplemented DMEM (Gibco). To produce LNCaP-eGFP cells, HEK293T were transfected using polyethylenimine (PEI) with packaging constructs (pMDLg/pRRE, pRSV-Rev, pCMV-VSV-G, AddGene). Virus was harvested after 24 h, filtered with a 0.22 µm filter (Millipore) and snap frozen in liquid nitrogen. LNCaP cells were infected at a MOI > 2 and selected with 2 µg/mL puromycin (Sigma) and checked for eGFP expression regularly.

### Three cell type co-culture assays

For co-culture assays, LNCaP cells and BJ fibroblasts were cultured together with either M1- or M2-like macrophages (Supplementary Fig. 1). Additionally, LNCaP cells were cultured with BJ fibroblasts, M1- or M2-like macrophages separately. Firstly, 3750 THP-1 cells were seeded in a 96-well plate (CELLSTAR plate, 96w, F, *ν*Clear, TC, PS, black, lid, Greiner) in 100 µL medium per well. THP-1 cells were differentiated towards M1- or M2-like macrophages following the above-mentioned protocol. LNCaP-eGFP cells were added to differentiated macrophages with or without BJ fibroblasts (4:1 ratio). To investigate the effect of different hormone conditions on LNCaP cell survival, all cells were cultured in 5% DCC and 1% PS RPMI-1640 and stimulated with either DMSO (vehicle) or 10 nM R1881. Additionally, cells were individually stimulated with either DMSO or 10 nM R1881 for 24 h, washed and co-cultured subsequently. LNCaP-eGFP cell fluorescence and proliferation was measured using IncuCyte Zoom (Essen BioScience) for 7 days. BJ fibroblast proliferation was measured separately by IncuCyte Zoom phase-contrast analysis. For illustrative purposes and to compare spatial patterns to in silico and in vivo patterns, we also cultured LNCaP cells and BJ fibroblasts once in a 1:1 ratio.

### Hormone conditions, apoptosis and resistant cell assays

To validate PCABM predictions on ADT effects, 3750 THP-1 cells were differentiated into M1- and M2 macrophages as described earlier in 5% DCC, 1% PS RPMI-1640. M1- and M2 macrophages were subsequently stimulated with either DMSO (vehicle) or 10 nM R1881 for 24 h. LNCaP-eGFP cells were seeded at a density of 15,000 cells per well in a 96-well plate (CELLSTAR plate, 96w, F, νClear, TC, PS, black, lid, Greiner) 24 h before the start of the assay in 100 µL of 5% DCC, 1% PS RPMI-1640 and were either stimulated with DMSO or 10 nM R1881 for 24 h. All cells were gently washed with PBS and LNCaP-eGFP cells were co-cultured in DMSO with either 3750 DMSO- or 3750 R1881 stimulated M1- or M2-polarized macrophages. Cell proliferation was measured with the IncuCyte Zoom fluorescent signal imaging system for 7 days. Data was normalized to time point zero (t = 24 h) to account for possible fluorescence intensity artifacts upon initialization. To compare Incucyte results to in silico results, PCABM data was normalized to the number of tumor cells upon initialization.

Cell apoptosis was measured and analyzed using IncuCyte Zoom (EssenBioScience) on similar cell numbers, timespans and set-up as described previously with 0.5 mM Caspase-3/7 Red Reagent for Apoptosis (Essen BioScience), while apoptosis control was induced by supplementing to 0.5 mM Phenylarsine Oxide (PAO, Sigma). To investigate growth of LNCaP-abl cells in androgen-deprived conditions, 250 LNCaP-abl cells were seeded on a 96-well plate and cultured in RPMI-1640, 5% DCC + 1% PS. Cell proliferation was measured and analyzed by brightfield analysis with the IncuCyte Zoom (Essen BioScience) for 10 days.

### Agent based model design

Our two-dimensional PCABM consists of four agents (cell types): tumor cells, M1 and M2 polarized macrophages, and fibroblasts as these are the most abundant cell types and key players in the prostate TME^62^. PCABM requires specific size grid cells, although in reality actual cell sizes vary, therefore each grid cell was assigned the size of one tumor cell^64^ as 142.89 µm^2^. Agents occupy exactly one position on a customizable rectangular grid, which size was scaled to in vitro well size leading to a 125 × 125 square grid (reality: 1.48 mm^2^).

To emulate in vitro settings, different agent types are randomly scattered on the grid upon initialization, with seeding densities matching in vitro experiments. PCABM runs for a fixed number of time steps of four hours every simulation, and each cell type has a probability to perform actions in the order: tumor cells, fibroblasts, M1 macrophages, and M2 macrophages (summarized in Fig. 1a).

Tumor cells can proliferate (TU_pprol_), die (TU_pdeath_, spontaneous death) or migrate (TU_pmig_) either towards fibroblasts or in random directions (TU_rwalk_) and have limited proliferation capacity (TU_pmax_). Fibroblasts can proliferate (F_pprol_) with limited capacity (F_pmax_), die (F_pdeath_, spontaneous) or migrate (F_pmig_) either towards tumor cells or in random directions (F_rwalk_). M1 and M2 polarized macrophages can migrate (M_pmig_) either towards tumor cells or randomly (M_rwalk_). Macrophages can kill (M_pkill_) when bordering a tumor cell, with maximum killing capacity (M_kmax_) before exhaustion and can spontaneously die (M_pdeath_). M2 polarized macrophages were calibrated to have attenuated tumoricidal activity compared to M1 polarized macrophages. Additionally, M2 polarized macrophages have the ability to increase tumor cell proliferation probability (M2_TUadd_).

Migration and proliferation processes requires unoccupied grid space in all agents’ neighborhood (Moore neighborhood), such that agents compete for space upon performing actions. Finally, inactive agents idle. All actions have calibrated stochastic probabilities, which resembles stochasticity observed in biological processes. The stochastic process works by drawing a random number between zero and one in each round for each agent. If the number is less than the agent’s stochastic action probability, the action is performed. An overview of model parameters is shown in Table 1.

### Initial parameter estimation

Tumor cell and macrophage migration parameters from Kather et al.^46,47^ were scaled to match PCABM grid size and time steps. Other parameter values were estimated using PSO, which uses swarm behavior to search for global solutions^65^ and has been useful in a variety of optimization problems, including ABM^46,66–68^. We ran PSO through Matlab using the mean squared error as cost function to search for local parameter minimum that best fit the in vitro data.

Relative tumor cell numbers produced by PCABM were compared to in vitro relative growth curves to estimate parameters. TU_pmax_ was assumed to be the same in presence or absence of hormone and estimated only in hormone proficient conditions, in which ADT is assumed to be non-toxic. TU_pprol_ was instead fitted independently in the two hormonal conditions. The tumor cell apoptotic probability was measured in vitro using a caspase 3 and 7 assay and was assumed equal for both androgen pro- and deficient conditions. PSO was ran 50 times for each biological replicate (replicate optimizations in Supplementary Fig. 2), with fixed parameter set to the median of the triplicate to be used as input for the next PSO iteration.

Similar to tumor cells, relative fibroblast numbers produced by PCABM were compared to relative fibroblast growth curves in vitro and parameters F_pprol_, F_pmax_ and F_pdeath_ were fitted. Fibroblast parameters were only optimized for DCC + R1881 conditions, since fibroblasts exhibit similar growth curves in androgen pro- and deficient conditions (Supplementary Fig. 2)^21^. Fibroblast migration parameters (F_pmig_ and F_rwalk_) and tumor cell migration towards fibroblasts (TU_rwalk_) were qualitatively tuned by comparing model visualizations to in vitro captured cell dynamics.

Macrophage optimizations were performed separately for M1- and M2-polarized macrophages in the presence of both tumor and fibroblast cells for both DMSO and R1881 conditions (Supplementary Figs. 3 and 4). Again, PCABM relative tumor cell numbers in macrophage presence were fitted to in vitro relative tumor cell numbers. The parameters M1_pkill_ and M1_kmax_ were optimized in hormone proficient conditions and killing capacities were assumed at maximum in these conditions as justified by our in vitro killing observations (Supplementary Fig. 5). However, for vehicle conditions only M1_pkill_ was optimized, as this value is reasonably lower in hormone deficient conditions. Similarly, M2-polarized macrophage killing M2_pkill_ was optimized with M2_kmax_ the same as M1_kmax_, although simultaneously M2_TUadd_ was optimized as tumor promoting growth parameter (Supplementary Fig. 5). A full list of the estimated parameters can be found in Table 1.

### Exploring effects of ADT on the prostate TME

Simulations solely included tumor cells and macrophages to exclude possible confounding effects of fibroblasts. Parameters were estimated similarly to previous parameter optimizations, optimizing 50 times with PSO in triplicate. However, instead of fixing the median parameter value over all triplicates to create one model, median parameters were fixed for each triplicate model individually. Killing probability (M_pkill_) and capacity (M_kmax_) of macrophages were estimated separately for M1- and M2-macrophages in hormone proficient conditions.

### Modeling castration resistance

Using PCABMs optimized hormonal TME conditions, CRPC growth was simulated by seeding a co-culture of androgen sensitive and resistant tumor cells (1:100) in hormone deprived conditions. Resistant tumor cells have different proliferation probability and capacity parameters (TU_pprolres_ and TU_pmaxres_ respectively), which were fitted using PSO to in vitro growth of LNCaP-abl cells (androgen ablated), an ADT resistant clone derived from LNCaP cells (Supplementary Fig. 2C). Resistant tumor cells migrate as fast as non-resistant cells and have the same probability of spontaneous death as non-resistant tumor cells in hormone proficient conditions. To simulate interactions in the TME upon CRPC development also fibroblasts, M1- or M2-macrophage agents were added. Since the amount of TME cell infiltration varies in prostate tumors, simulations were run with various ratios of different cell types.

### Patient samples and histology

Spatial cellular patterns produced by PCABM were compared with a histological sample from a radical prostatectomy specimen, which was formalin fixed, paraffin embedded (FFPE). Tissue was stained with H&E and a 200× enlarged microscopy image was taken.

The use of patient archival prostatectomy materials for research purposes at the Netherlands Cancer Institute, have been executed pursuant to Dutch legislation and international standards. Written informed consent was obtained from all patients.

### Statistical analysis

Statistical analysis of growth rate differences in hormone conditions was performed using linear mixed-effect models with longitudinal analysis using R package *TumGrowth*^69^.For validation, in vitro LNCaP cell growth was tested in different hormone conditions over time and also PCABM output for CRPC simulations with different cell types was analyzed similarly. Different TME compositions were tested for effects on simulated relative tumor cell number over time. Type II analysis of covariance (ANOVA) with Wald tests were used to calculate *p*-values with significance cutoff 0.05.

### Reporting summary

Further information on research design is available in the Nature Research Reporting Summary linked to this article.

### Supplementary information

Supplementary Material

Reporting summary

## Data Availability

Cell culture data are deposited in Zenodo 10.5281/zenodo.10517653.

## Code availability

The model used in this study is publicly available in GitHub https://github.com/SysBioOncology/PCABM_ADT.
