# Supplementary material for: Agent-based modeling of the prostate tumor microenvironment uncovers spatial tumor growth constraints and immunomodulatory properties
Source: NPJ Syst Biol Appl. 2024 Feb 21;10:20. doi: 10.1038/s41540-024-00344-6 (PMC10881528; doi:10.1038/s41540-024-00344-6)
Supplement: Supplementary file 1 — Supplementary Material [file 41540_2024_344_MOESM1_ESM.pdf]

Supplementary Files

Agent-based modeling of the prostate tumor microenvironment uncovers spatial tumor growth constraints and immunomodulatory properties

Maisa van Genderen, Jeroen Kneppers*, Anniek Zaalberg*, Elise Bekers, Andries M Bergman†, Wilbert Zwart†, Federica Eduati†*

**These authors have contributed equally*

†To whom correspondence can be addressed: Federica Eduati f.eduati@tue.nl, Andre Bergman a.bergman@nki.nl and Wilbert Zwart w.zwart@nki.nl

Supplementary Figures 1-8

Supplementary Table 1

Supplementary Figure 1

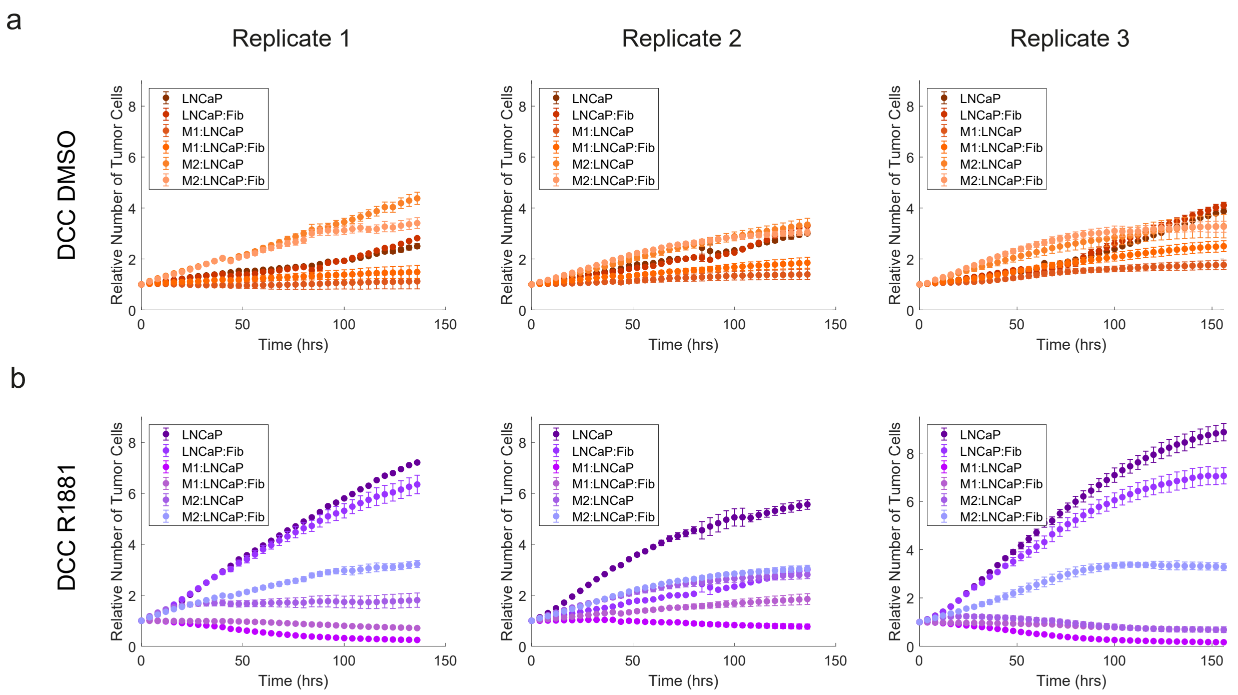

Supplementary Figure 1: Biological replicates of LNCaP co-cultures in hormone deficient and proficient conditions.

a) Three biological replicates of LNCaP cells co-cultured with fibroblasts alone, M1-or M2-macrophages alone or co-cultured with both cell types in hormone deprived conditions.

b) Three biological replicates of LNCaP cells co-cultured with fibroblasts alone, M1-or M2-macrophages alone or co-cultured with both cell types in hormone proficient conditions.

Dots represent the average of six technical replicates. Error bars represent the standard deviation.

Supplementary Figure 2

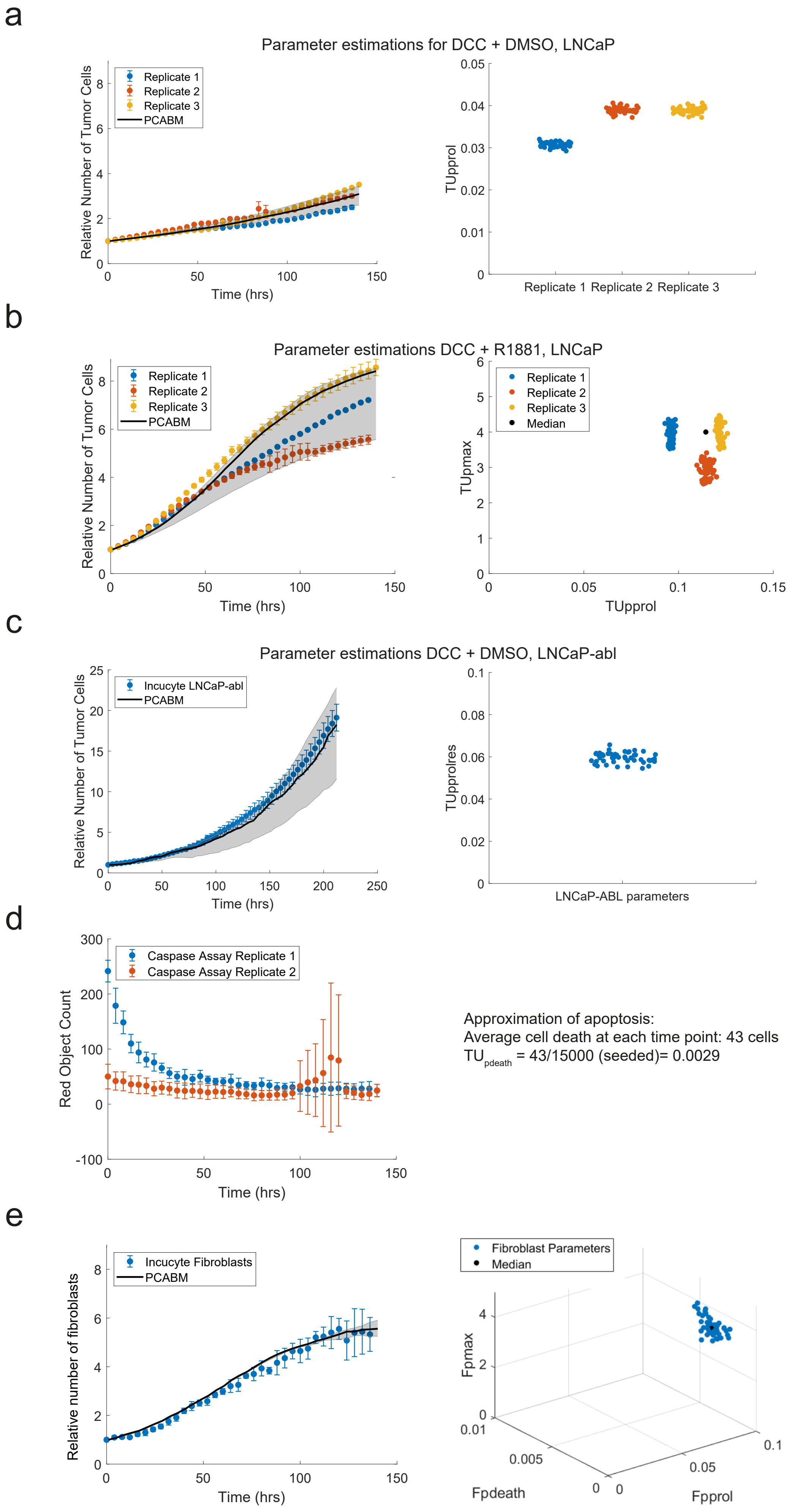

Supplementary Figure 2: Particle Swarm Optimization estimations of parameters for LNCaP cell growth, apoptosis and LNCaP cell growth co-cultured with fibroblasts.

a) Three biological replicates of LNCaP growth curves in hormone deficient conditions (left, dots represent Incucyte data, solid line represents PCABM). Estimation of the parameter TU_{pprol} in hormone deficient conditions using three biological replicates (right).

b) Three biological replicates of LNCaP growth curves in hormone proficient conditions (left, dots represent Incucyte data, solid line represents PCABM). Estimation of parameters TU_{pprol} and TU_{pmax} in hormone proficient conditions using three biological replicates (right).

c) LNCaP-abl growth data (Incucyte) overlain with PCABM output with optimized parameters (left). Estimation of TUpprolres (right).

d) Incucyte red object count for caspase 3/7 assay with LNCaP cells. An object is interpreted as a dying cell.

e) Fibroblast growth data (Incucyte) projected on PCABM output with optimized parameters (left). Estimation of F_{pmax}, F_{pdeath} and F_{pprol} (right).

Optimized parameter sets (50 optimizations) for each biological replicate. For Incucyte data, dots represent the mean and error bars represent the standard deviation of six technical replicates. For PCABM data, lines represent *in silico* model output with median optimized parameters. Shading represents model output for optimized parameters within interquartile range for 50 optimizations per biological replicate.

Supplementary Figure 3

a

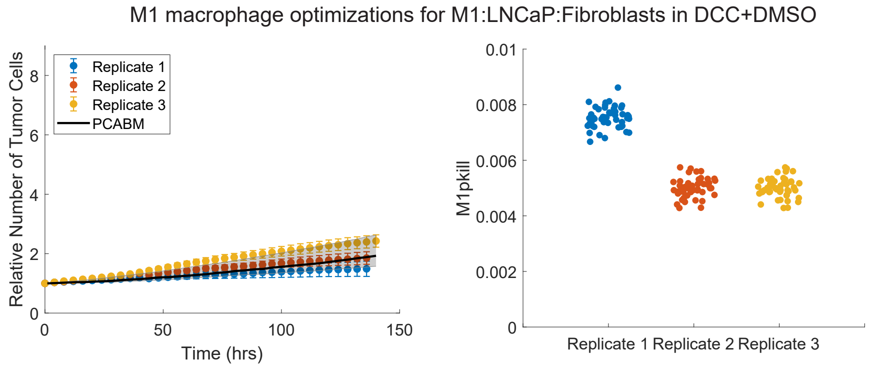

b

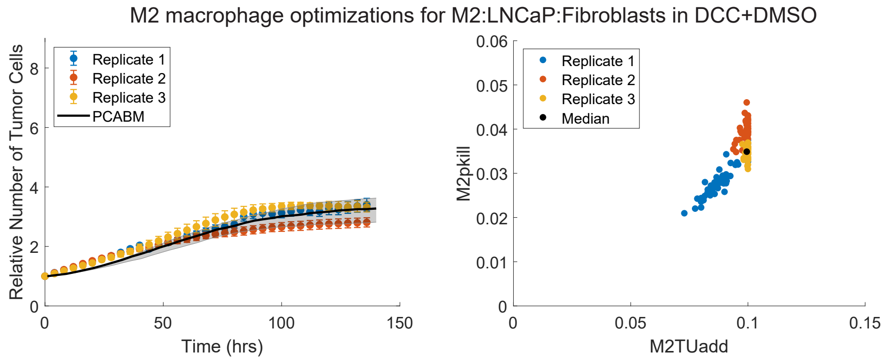

Supplementary Figure 3: PCABM parameter estimations for LNCaP cells co-cultured with fibroblasts and M1-or M2-polarized macrophages in hormone deficient conditions.

a) Three biological replicates of growth curves of LNCaP cells co-cultured with fibroblasts and M1 macrophages is hormone deficient medium (left, dots represent Incucyte data, solid line represents PCABM). Estimation of $M1_{pkill}$ in hormone deficient conditions (right).

b) Three biological replicates of growth curves of LNCaP cells co-cultured with fibroblasts and M2 macrophages is hormone proficient medium (left, dots represent Incucyte data, solid line represents PCABM). Estimations of $M2_{pkill}$ and $M2_{TUadd}$ in hormone deficient conditions (right).

Optimized parameter sets (50 optimizations) for each biological replicate.

For Incucyte data, dots represent the mean and error bars represent the standard deviation of six technical replicates. For PCABM data, lines represent *in silico* model output with median optimized parameters. Shading represent model output for optimized parameters within interquartile range for 50 optimizations per biological replicate.

Supplementary Figure 4

a

M1 macrophage optimizations for M1:LNCaP:Fibroblasts in DCC + R1881

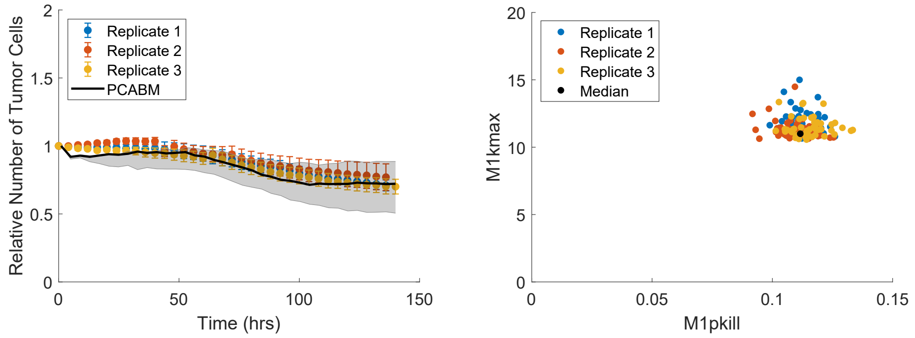

b

M2 macrophage optimizations for M2:LNCaP:Fibroblasts in DCC+ R1881

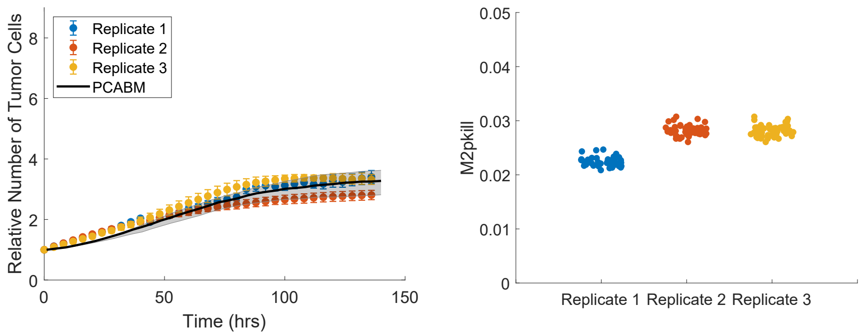

Supplementary Figure 4: PCABM parameter estimations for LNCaP cells co-cultured with fibroblast and M1-or M2-polarized macrophages in hormone proficient conditions.

a) Three biological replicates of growth curves of LNCaP cells co-cultured with fibroblasts and M1 macrophages is hormone proficient medium (left, dots represent Incucyte data, solid line represents PCABM). Estimations of $M1_{pkil}$l and $M1_{kmax}$ in hormone proficient conditions (right).

b) Three biological replicates of growth curves of LNCaP cells co-cultured with fibroblasts and M2 macrophages in hormone proficient medium (left, dots represent Incucyte data, solid line represents PCABM). Estimations of $M1_{pkill}$ and $M1_{kmax}$ in hormone proficient conditions (right).

Optimized parameter sets (50 optimizations) for each biological replicate. For Incucyte data, dots represent the mean and error bars represent the standard deviation of six technical replicates. For PCABM data, lines represent *in silico* model output with median optimized parameters. Shading represent model output for optimized parameters within interquartile range for 50 optimizations per biological replicate.

Supplementary Figure 5

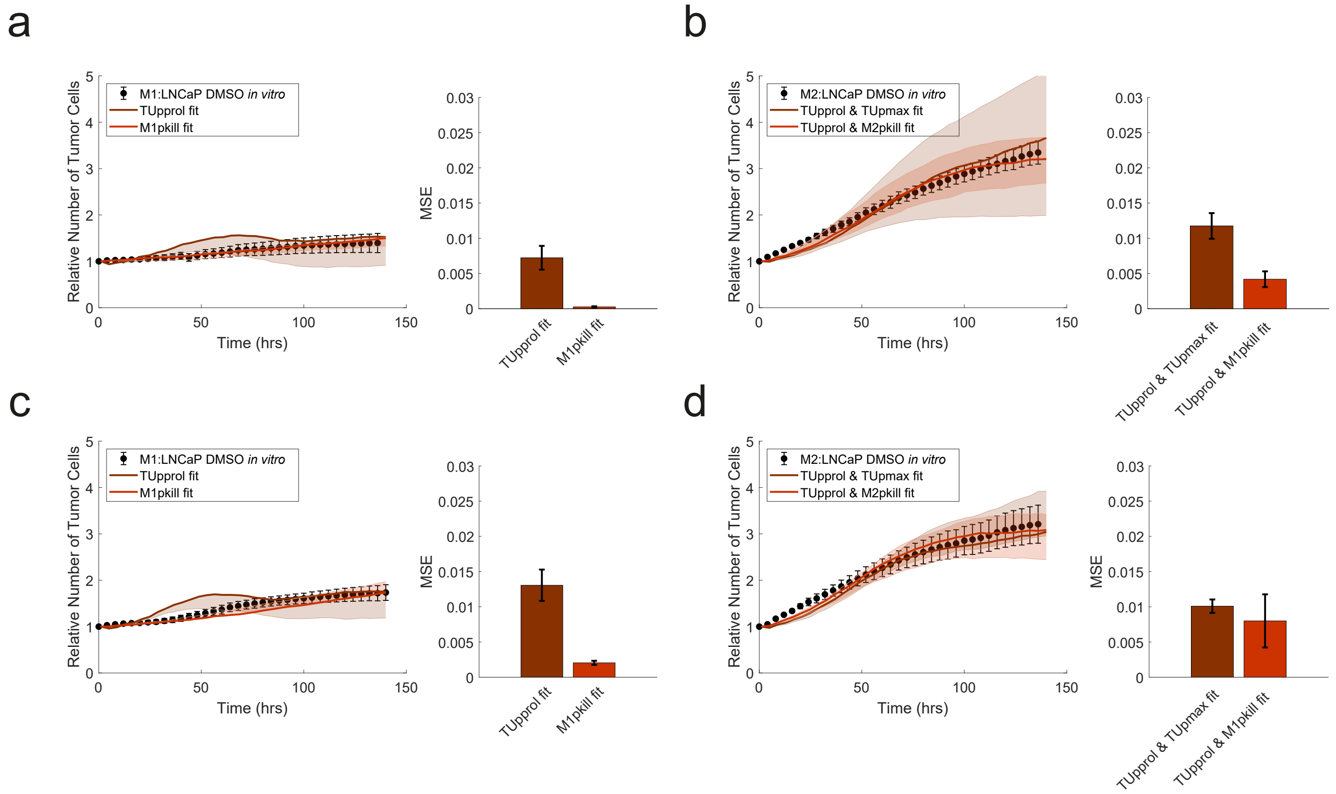

Supplementary Figure 5: PCABM optimization for macrophage conditions.

a) Optimization replicate 2 M1:LNCaP. PCABM optimization for TU_{pprol} and $M1_{pkill}$ in DCC+DMSO (left) and mean squared error (MSE) between data and PCABM for M1:LNCaP TU_{pprol} and $M1_{pkill}$ (right).

b) Optimization replicate 2 M2:LNCaP. PCABM optimization for TU_{pprol} + TU_{pmax} and TU_{pprol} + $M2_{pkill}$ in DCC DMSO (left) and mean squared error (MSE) between data and PCABM for M2:LNCaP TU_{pprol} + TU_{pmax} and TU_{pprol} + $M2_{pkill}$ (right).

c) Optimization replicate 2 M1:LNCaP. PCABM optimization for TU_{pprol} and $M1_{pkill}$ in DCC+DMSO (left) and mean squared error (MSE) between data and PCABM for M1:LNCaP TU_{pprol} and $M1_{pkill}$ (right).

d) Optimization replicate 2 M2:LNCaP. PCABM optimization for TU_{pprol} + TU_{pmax} and TU_{pprol} + $M2_{pkill}$ in DCC DMSO (left) and mean squared error (MSE) between data and PCABM for M2:LNCaP TU_{pprol} + TU_{pmax} and TU_{pprol} + $M2_{pkill}$ (right).

Dots represent average and error bars represent standard deviation of six technical replicates. Lines represent PCABM output with the median of optimized parameters. Shading represents model output for optimized parameters within interquartile range given by 50 optimizations.

Supplementary Figure 6

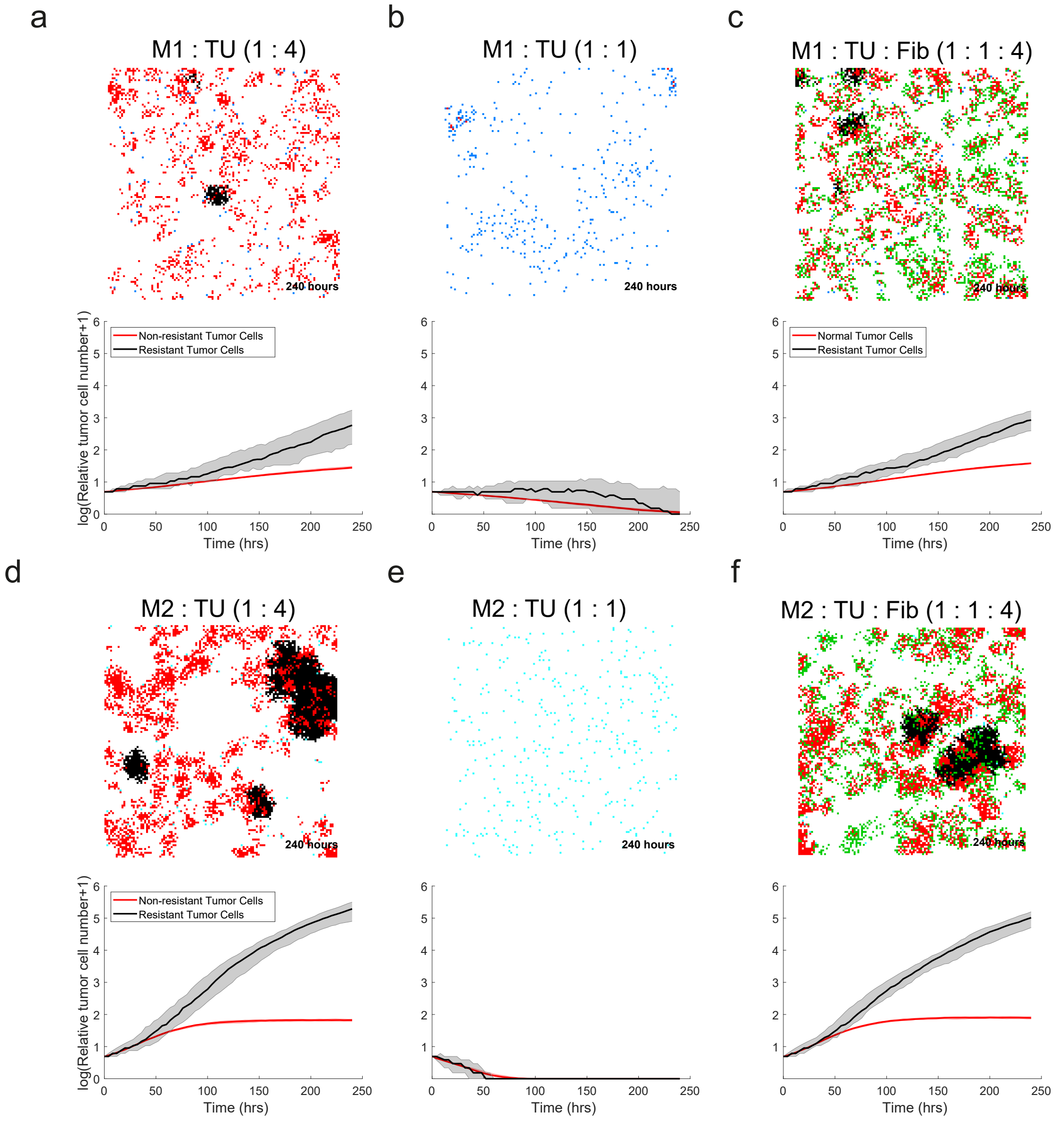

Supplementary Figure 6: CRPC simulations

a) LNCaP cells co-cultured with M1 macrophages at a 1:4 ratio

b) LNCaP cells co-cultured with M1 macrophages at a 1:1 ratio

c) LNCaP cells co-cultured with M1 macrophages and fibroblasts at a 1:4:4 ratio

d) LNCaP cells co-cultured with M2 macrophages at a 1:4 ratio

e) LNCaP cells co-cultured with M2 macrophages at a 1:1 ratio

f) LNCaP cells co-cultured with M2 macrophages and fibroblasts at a 1:4:4 ratio

LNCaP-abl cells are present in all simulations and are seeded with LNCaP cells at a 1:100 ratio. The initial amount of tumor cells does not change throughout the different simulations. Upper panels: LNCaP cells are shown in red, LNCaP-abl cells are shown in black, fibroblasts are shown in green and macrophages are shown in blue. Simulated growth after 240 hours (60 iterations) is shown.

Lower panels: lines and shading represent the median and interquartile range of 50 simulations. Red lines are relative growth for LNCaP cells and black lines are relative growth for LNCaP-abl cells.

Supplementary Figure 7

Co-culture
M1:LNCaP:LNCaP-abl:fibroblasts

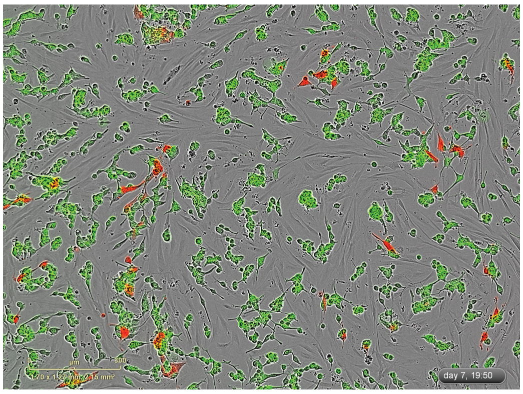

300 uM

Co-culture
M2:LNCaP:LNCaP-abl:fibroblasts

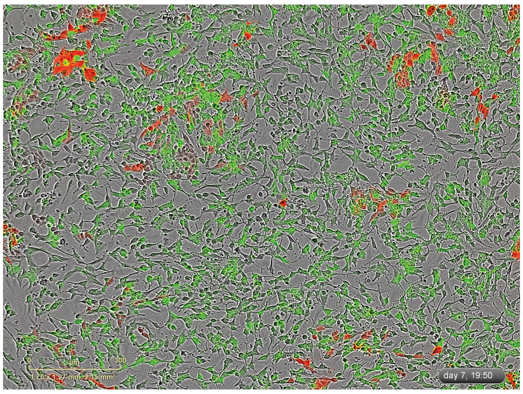

300 uM

Supplementary Figure 7: *In vitro* co-cultures of CRPC

Left panel: co-cultures with M1 macrophages, LNCaP cells and Fibroblasts in a 1:4:1 ratio. Right panel: co-cultures with M2 macrophages, LNCaP cells and Fibroblasts in a 1:4:1 ratio.

In both panels LNCaP-abl cells are seeded with LNCaP cells in a 1:100 ratio. LNCaP-abl cells are shown in red and LNCaP cells are shown in green.

Supplementary Figure 8

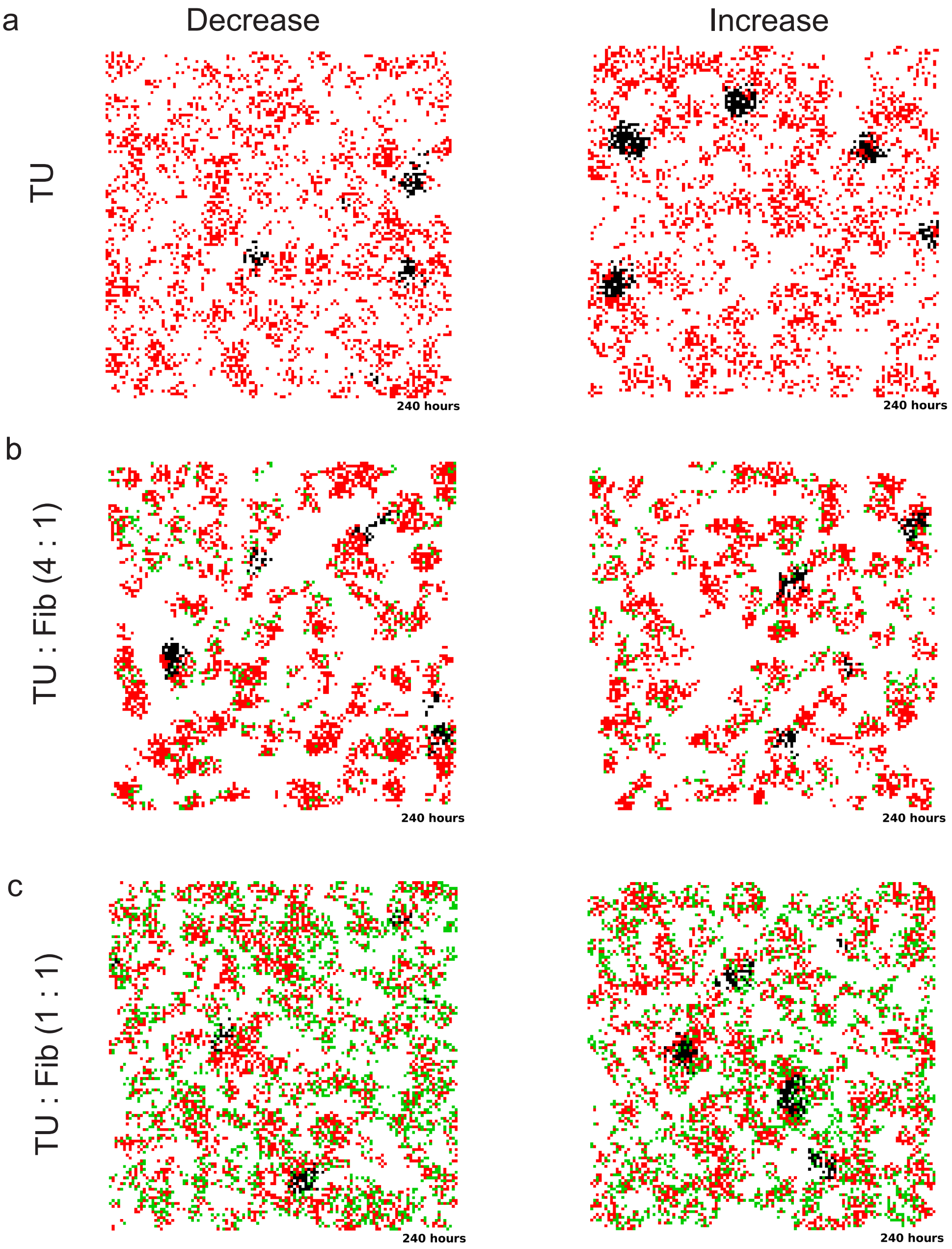

Supplementary Figure 8: CRPC parameter variations

a) Spatial growth patterns of LNCaP and LNCaP-abl cells with a 10% decrease (left) or increase (right) of LNCaP-abl *TUpprolres* and *TUpmigres* parameters.

b) Spatial growth patterns of tumor cells seeded with fibroblasts at a 4:1 ratio with a 10% decrease (left) or increase (right) of LNCaP-abl TUpprolres and TUpmigres parameters.

c) Spatial growth patterns of tumor cells seeded with fibroblasts at a 1:1 ratio with a 10% decrease (left) or increase (right) of LNCaP-abl TUpprolres and TUpmigres parameters.

LNCaP-abl cells are present in all simulations and are seeded with LNCaP cells at a 1:100 ratio. The initial amount of tumor cells does not change throughout the different simulations. LNCaP cells are shown in red, LNCaP-abl cells are shown in black, fibroblasts are shown in green and macrophages are shown in blue. Simulated growth after 240 hours (60 iterations) is shown. The increase and decrease of parameters was compared to the optimized parameter set and simulations as shown in Figure 5 and Supplementary Figure S2C.

Supplementary Table 1: Parameter optimization for macrophage hormone conditions across triplicates

Replicate	Condition	Parameters (median)
**1**	M1:LNCaP DMSO	TUpprol = 0.1434; M1pkill = 0.0096
**1**	M1:LNCaP R1881	M1pkill = 0.2006; M1kmax = 12
**1**	M2:LNCaP R1881	M2pkill = 0.0408; M2kmax = 12
**1**	M2:LNCaP DMSO	TUpprol = 0.1338; TUpmax = 5
**1**	M2:LNCaP DMSO	TUpprol = 0.0973; M2pkill = 0.0109
**2**	M1:LNCaP R1881	M1pkill = 0.1815; M1kmax = 12
**2**	M1:LNCaP DMSO	TUpprol = 0.1530; M1pkill = 0.0072
**2**	M2:LNCaP R1881	M2pkill = 0.0434; M2kmax = 11
**2**	M2:LNCaP DMSO	TUpprol = 0.1293; TUpmax = 5
**2**	M2:LNCaP DMSO	TUpprol = 0.1118; M2pkill = 0.0218
**3**	M1:LNCaP DMSO	TUpprol = 0.1684;M1pkill = 0.0051
**3**	M1:LNCaP R1881	M1pkill = 0.2297; M1kmax = 12
**3**	M2:LNCaP R1881	M2pkill = 0.0777; M2kmax = 13
**3**	M2:LNCaP DMSO	TUpprol = 0.1510; TUpmax = 5
**3**	M2:LNCaP DMSO	TUpprol = 0.1240; M2pkill = 0.0281
